# Some Medical Aspects of the Pan-American Exposition

**Published:** 1901-08

**Authors:** 


### Some Medical Aspects of the Pan-American Exposition.

(*From Boston Medical and Surgical Journal, July 18 and 25, 1901.*)

THE Pan-American Exposition presents certain features of particular interest to medical visitors, which, however, are so widely scattered that many of them are overlooked by the casual observer, who has no official guide to their location. Hence a brief mention of their whereabouts and special points of interest may be of value.

The Emergency Hospital, supported from the funds of the exposition and treating its cases gratuitously, is located immediately on the right of the West Amherst street gate, through which the majority of visitors enter the exposition grounds. It is an artistic little building of cream-colored staff, topped off with a dull red roof. It was erected early in the process of constructing the fair, to meet the medical and surgical emergencies contingent on the presence of a large number of workmen, many of them engaged in more or less hazardous callings. Its functions are confined purely to emergency work, and any severe cases received during the day are transferred to the Buffalo General Hospital, or other points outside the grounds, if prolonged treatment is required. No cases are allowed to remain over night in the Emergency Hospital, and no venereal cases are treated therein. The institution has a capacity of 26 beds, and includes a small dispensary fitted up with a few simple remedies, a surgical dressing-room, an excellent little operating-room, and a small diet kitchen. It is admirably administered under the supervision of Dr. Roswell Park, the Medical Director of the Exposition, whose surgical abilities are well known to the profession, assisted by Dr. Vertner Kenerson, both of whom visit the institution one or more times daily. The personnel of the hospital consists of six young physicians who act as the house staff, and two of whom are constantly on duty. Four nurses constitute the nursing staff. The hospital supports an ambulance service, consisting of one automobile ambulance, the drivers of which are medical students from the University of Buffalo.

Up to the present time, the hospital has treated about 2,200 cases, of which about 700 were surgical. At present most of the cases requiring attention are of a medical nature and are of trivial character, about 30 cases being treated daily. The majority of these cases are of a diarrheal nature and are furnished by the employees of the large foreign concessions in the Midway, such as the "Streets of Cairo," "Indian Congress," "Filipino Village," etc. One member of the house staff usually visits these larger concessions daily, to ascertain the presence of any cases needing medical attention. So far, the professional work has been very light. A few deaths and seven accidents occurred as a result of falls by workmen during erection of the exposition buildings, and, as was to be expected in a fair where electricity is made such a prominent feature, several fatal accidents among the workmen have occurred from contact with defectively insulated electric conduits. Scarcely any cases of sunstroke and heat exhaustion have as yet occurred, even during the hot wave which recently prevailed over the eastern part of the United States. Cool lake breezes are a pleasant feature of Buffalo summer climate and seem to substantiate the claim that Buffalo is the coolest city in the Union during the summer. There has not been a night this season when the visitor would not require the use of a blanket to sleep in comfort. Mosquitoes and flies are almost unknown in Buffalo, and the visitor is certain to be free from their annoyance. To return to the subject of the Emergency Hospital, it may be said that while this institution possesses no unusual features to those acquainted with hospital service, its equipment is of the most modern type, and its neatness and order, together with the efficacy of its service, reflect much credit upon those in charge.

### INFANT INCUBATORS.

A few steps from the Emergency Hospital is a building which somewhat resembles this institution in appearance. It is the building of the "Infant Incubators," which a "barker," in high hat and frock coat terms "the only scientific attraction on the Midway. The Qbata Company, which controls this concession, appears to be coining money, for, strange as it may seem, this attraction is one of the most popular on the Midway. Its patrons are not only those who have a professional interest in the subject, but also a large proportion of the curious, particularly those of the feminine persuasion. As the "barker" says, "there is nothing improper in the exhibit," but his statement that there is "a whole houseful of infants," must be taken with the usual Midway grain of salt. Inside, the visitor is ushered into a large room, with impermeable walls and floor, railed off around the walls. There are about a dozen of the Qbata incubators, something more than half of which are usually occupied by small mites of humanity prematurely introduced to the world. The incubator consists of a nickel frame with glass sides, making a box about two feet square, to which access is had by a door in front. The infant neatly bundled up, lies on a small mattress supported on springs so delicate that the whole frame gently oscillates with every movement, even the breathing of the child.

The temperature in the incubator is regulated by a thermostat, which maintains a constant temperature of about 26° C., the necessary warmth being supplied from water pipes running from a small tank heated by an oil lamp. Ventilation of the incubators is secured by small aspirating fans, the incoming air being rendered sterile by filtration through cotton. Wet nurses provide nature's food for the sustenance of the infants, the latter being removed from the incubators and fed every two hours, weighed, and the cleanly condition of their linen verified. This is done in a small nursery, adjoining the incubator room, which is fitted up with bath tubs, baby baskets, and the like, in a way to satisfy aseptic ideas and delight the esthetic. A chart on each incubator shows the age, sex, weight, temperature and period of gestation of each child. One small morsel of humanity on exhibition was stated to have weighed 2 lbs., 4 oz. on entrance and to have gained 5 oz. in about a fortnight's sojourn in the incubator. The period of gestation of nearly all the infants is stated as about seven months. The appearance of some of the infants would appear to justify this statement, though it would require a slight elasticity of the professional imagination to believe this with regard to others. The supply of infants is recruited from Buffalo and vicinity, they being cared for gratuitously by the Qbata Company. I was informed that a premature arrival in a family prominent in Buffalo society and a small pappoose from the Indian Congress were admitted on the same day.

The Qbata Company claims that 85 per cent. of viable infants may be saved by their incubators. The question naturally presents itself as to whether this is worth while; whether the race as a whole does not suffer from the preservation of these weaklings to perpetuate their kind. Medical science is a little illogical in respect to the results obtained, and in its efforts to preserve the individual it forgets to consider the effects of such action upon the race as a whole. Every stock raiser appreciates the necessity of healthful environment, abundant food and fresh air in maintaining a breed of animals in a state of high physical development; and sanitary science insists upon the necessity of these conditions for the physical uplifting of the human race. The stock raiser, however, breeds only from the most sound, healthy and perfect animals, and thus secures a physical conformation and constitution upon which conditions of environment can act most advantageously. Medical science, on the other hand, does not hesitate to undo the advantages gained by the hygienic rules it has promulgated, by preserving the weakling, the deformed and the tuberculous, and placing these defectives—who would otherwise surely have perished in an active struggle for existence—in a condition to transmit their deficiencies, deformities and vices to generations as yet unborn. Certainly, in regard to the physical standard of the human race, the medical profession is in the position of tearing down with one hand while it builds up with the other.

On the whole, the exhibit of infant incubators furnishes much food for reflection and is well worth the cost of admission. The concession is well cared for and everything about it is kept neat, clean and attractive, and elicits the commendation of visitors in this respect. I suspect that the general opinion in regard to the infants themselves is fairly well expressed by the Englishman whom I overheard remark as he emerged from the incubator room, "Only fancy commencing life as a Midway exhibit, don't you know!"

---

### A CRECHE NEEDED.

In my last letter a description was given of the Exposition Emergency Hospital and the "infant incubators" on the Midway. From the latter subject to that of the care of young children accompanying older persons visiting the fair the transition is easy. At present a "crèche," where tired mothers may safely leave their children, is much needed.[1] The Children's Building at the south end of the Midway, originally intended for this purpose, has been converted into headquarters for representatives

1. A Crèche has been established.—Ed. B. M. J.

of the press, for which latter purpose its location and size render it particularly suitable. There has been some talk of establishing nursery tents at each of the main entrances to the grounds, but as yet nothing has been done in this respect, nominally from lack of funds. The matter is one which merits the serious attention of the exposition authorities, and any additional cost resulting from the operation of these nurseries would probably be more than repaid by an increase in the gate receipts. With the poorer classes the mother frequently cannot take an outing unless accompanied by her children, from lack of anyone who can be relied upon to care for them during her absence. If the exposition provided places where infants might be safely left, the fair would undoubtedly be patronised to a greater extent by mothers of this class. Sightseeing on a warm day while carrying a baby in arms is not conducive to enjoyment on the part of the tired and overworked mother nor good temper and health on the part of the unfortunate infant exposed to the heat, noise and crowds.

Crossing the Midway the average medical visitor proceeds to the beautiful court of fountains, and turns his steps toward the exhibition made by various departments of the government, well called the "back-bone of the exposition." On his way he will very likely visit the Ethnology Building, which contains an ethnological display which, though not large, is of unusually high character. In this building the medical men interested in anthropology will find a certain display on this subject worthy of attention, chiefly among the exhibits in the gallery.

### PURE FOOD EXHIBIT.

In the exhibit of the Department of Agriculture, located next the Ethnology Building, in the annex on the right of the main Government Building, the display made by the section on foods will prove of much interest to the medical man. This exhibit is in charge of Dr. Stewart of the Bureau of Animal Industry, and occupies one-fourth the floor space of the building, being located immediately west of the main entrance. Much space is devoted to the subject of adulteration of foods, and numerous specimens, tastefully arranged in jars, are used to illustrate the appearance of some of the more common articles of food, both pure and after adulteration by some of the more common methods. The exhibit is particularly rich in its display relative to the adulteration of spices. An interesting series of specimens illustrates the various grades of canned fruits and vegetables, from the highest quality down to the watered and reprocessed article billed by the dishonest tradesman as "first quality." The artificial coloration of various foods is shown; also the crude material from which these pigments are obtained, and the special forms in which they are employed commercially. One test tube shows 10 gr. of copper recovered from a single 1 lb. can of string beans. The possibility of tin poisoning is illustrated by 5 gr. of stannous oxide recovered from a 1 lb. can of tomato soup.

An excellent series of food preservation is shown in glass containers, each bearing the chemical analysis of the contained article and the actual market cost of the materials, together with the selling price. Such remarks as "formaldehyde solution; retail price, $1.00 per gallon; value of materials less than 4 cents," point their moral very concisely. One cannot but wish that the Government would follow the example of the German authorities in respect to patent medicines and proprietary nostrums, and publish the formulæ and ingredients of all such preparations, together with their cost of preparation at market rates for materials. The fortunes amassed by many of the concerns controlling patent medicines of certain therapeutic efficiency undoubtedly depend upon secrecy of preparation; for the hard-headed citizen can scarcely be expected to spend a dollar for a ready-made article when he can have the same thing put up at the corner drug-store for half the money. The enaction of a law requiring all manufacturers of patent medicines to print the formula upon the label is respectively submitted as worthy of the best efforts of the profession in respect to future legislation.

An interesting exhibit in connection with the artificial preservation of food is seen in a collection of tubes displaying quantities of salicylic acid and other substances recovered from small quantities of food stuffs preserved by their agency. Half a test tubeful of salicylic acid is shown as having been recovered from a single tin of canned soup—and one is moved to marvel that cases of poisoning from preserved food stuffs are not more common than they are. "Preservative,"—a combination of boric acid and salt, colored with cochineal,—made famous in the army beef controversy, is here given a prominent place. One of the exhibits among the jams and preserves is labelled: "Strawberry Jam." Sweetened with glucose, stiffened with starch, colored with an aniline dye, preserved with benzoic acid and artificially flavored." The strawberry part of this delectable compound apparently exists in the imagination alone. It is highly unfortunate that the exhibit does not specify the particular brands and give the manufacturers' names of the articles whose analysis are displayed, so that the observer might not only appreciate the extent to which food adulteration is practised, but might know what brands to avoid in making future purchases. Those whose greed is such as to render them willing to injure the public health to more quickly fill their purses should be publicly pilloried and made to suffer the financial loss which would follow exposure of their nefarious practises.

An interesting series of "wines," made by fermenting glucose, colored with aniline dyes and preserved with salicylic acid, is also on exhibition. It is stated that these "wines" are sold to the trade at from 25 cents to 35 cents per gallon.

A large case in the centre of the building contains a bomb calorimeter, used for the determination of the force or fuel value of food stuffs. The same case also contains a model of Atwater's respiration calorimeter, as used by him in his investigations on nutrition at Wesleyan University.

### MEAT EXAMINATION.

In the Bureau of Animal Industry, a feature which attracts the attention of crowds, is the microscopic examination of pork for trichinæ and other parasites, as carried out by the Department of Agriculture at the large packing houses. A small laboratory is here fitted up, in which three young women make these microscopical examinations in the presence of the visitors, and exhibit samples of infected meat. Nearby an interesting series of pathological specimens, both wet and artificial, showing various types and lesions of disease in the animals used as food, will prove interesting to all medical men, and is well worthy of careful study by health officers and those who have to do with food inspection. This exhibit is supplemented by a large series of lantern slides, showing bacteria, pathogenic lesions, etc.

Many artificial preparations illustrate the method of infection and pathologic changes in Texas fever, the investigations into the cause and nature of which by the Agricultural Department formed the entering wedge which opened the way to a wider knowledge of the transmission of disease by insects, the latest triumph of scientific medical research. A small but completely equipped biologic laboratory is also shown in this section; also various protective and curatives are, and illustrations of the steps in their preparation. The municipal health officer will find much to interest him in the nearby display of sanitary dairy cans, bottles and utensils; and the photographs, illustrating the sanitary methods now employed in the best class of dairies to minimise the danger of transmitting disease by their products.

Before leaving this exhibit the physician, who naturally has much to do with horses, will find it of advantage to inspect the case illustrating the causes of lameness in horses, proper and improper methods of shoeing, and the like. The knowledge to be obtained in a few minutes from this exhibit will be of much practical value, and may save suffering on the part of the animal and money and annoyance to the owner.

### MEDICAL DEPARTMENTS OF ARMY AND NAVY.

Proceeding through the connecting arcade to the main Government Building the professional visitor will find much to interest him in the exhibits made by the medical departments of the Army and Navy and of the Marine Hospital service.

The exhibit of the Medical Department of the Army—the largest single exhibit of any character in the entire exposition—consists of a model brigade field hospital, lack of suitable floor space in the Government Building having rendered any indoor display commensurate with the importance of the department, quite impossible. While the present exhibit is most admirable so far as it goes, it is to be regretted that the Army Medical Department, with its magnificent museum, Surgeon-General's library and completely equipped laboratories to draw upon for exhibits, did not have the opportunity of demonstrating its resources and the magnitude and value of its work by an indoor display. It is understood that but 400 feet of inside space was placed at the disposal of the department, so that probably the latter did wisely in refusing to make an indoor exhibit which could not be representative.

The brigade field hospital tents are located on the large plot immediately south of the arcade between the Fisheries and main Government Buildings, and are much visited, not only by physicians, nurses and military men, but also by a large class who have—or who have had—friends or relatives in the regular or volunteer armies, and are interested in the care of the sick soldier in the field. The hospital has a capacity of 100 beds—or a proportion of about 2 per cent. from a command of 5,375 maximum war strength—and is completely equipped for field service down to the last authorised dose of medicine and tent peg. The purpose of the exhibit is to leave nothing to the imagination of visitors, but to demonstrate the equipment of the medical department in respect to the brigade hospital unit, in quantity, size and capacity, as well as in variety and quality. The exhibit is peculiarly unique and attractive, since the equipment displayed is largely composed of the articles lately incorporated in the supply table of the medical department, as a result of the labors of a board of medical officers who were engaged for nearly two years on the improvement of the hospital equipment and medical supplies. Nearly all the important articles here shown have been adopted by the medical department within the past twelve months, and the exhibit as a whole undoubtedly represents a much more modern and complete equipment for the care of the sick and wounded in the field than could be shown by any other army in the world. Medical men will be most favorably impressed by this exhibit, with the resources and progressiveness of the Army Medical Department. The hospital tent wards of this exhibit are pitched in the form of a cross, with a central covered space. The medical and surgical tents, office, mess and kitchen tents are located within the arms of the cross, presenting an arrangement not only attractive and compact, but so devised as to afford the casual visitor a good idea of army medical service in the field with the minimum expenditure of time and effort.

The dispensary tents contain drugs and medicines in such quantities, varieties and proportions as military experience, since the outbreak of the war with Spain, has shown to be required for a brigade of maximum war strength, under conditions of field service, for a period of ninety days. The new model medical chest displayed in this tent is a marvel of simplicity and compactness, and should prove of special interest to medical officers of the state troops. Weighing only about eighty-five pounds, it yet contains an abundance of medicines and medical supplies for a regiment for three months. This chest forms one of the regimental set of three field chests,—the medical, surgical and steriliser,—one chest of which can be carried by a single coolie, two can be carried on a litter, and the whole three of which may be carried on a pack mule. The brigade hospital reserve supply of medicines is shown in bulk in the original bottles, but the hospital corps man in charge explained a simple method of packing such reserve medicines which would be used in the future; the method depending on the issue of medicines in bottles of standard sizes and shapes, four small bottles making a packet of the same proportions and size as one large bottle, and doing away with the necessity for the use of partitions or packing materials in the containing chests, to prevent breakage. The ward tents used in the exhibit are of the new model Munson hospital tent pattern, recently adopted by the medical department, as a result of exhaustive trial in the United States and in the tropics, as being much superior to the old hospital tents, formerly employed for the shelter of the sick. The wards are very cool and attractive, the tents are admirably ventilated, and the subdued light and seclusions these tents afford must be very grateful to the sick soldier. One is struck by the remarkable economy of space, transportation and labor possible by the new method of packing the hospital furniture and equipment. A complete outfit of cots, chairs and tables for each tent, allowance of six patients, goes in a single small chest, while all the bedding, pajamas, mosquito bars, and the like, pack in a single canvas bag—thus saving the necessity of opening numerous chests and boxes to secure the various articles necessary to outfit the tent on establishing the hospital. In the covered space between the four wards an extremely interesting series of photographs is displayed, illustrating actual field work of the medical department in transportation of wounded, first aid and field surgery, and hospital establishment.

---

It was a most excellent plan to establish "comfort stations" at available points, even of a temporary kind, and it was a senseless act to remove them because a few hyperesthetic individuals objected to them on account of their unsightliness. A large city like Buffalo, with the increased temporary population during the exposition period, would be guilty of a breach of civility did it not provide such accommodations, and the question of architectural beauty might well remain in abeyance until the emergency of the season had passed.

We do not attempt to defend the weak policy of spending several thousand dollars in establishing ugly temporary stations, when comely permanent ones should have been provided long ago. Our design is rather to point out not only the absurdity of listening to objectors to the temporary stations now established, but also the equally reprehensible policy of spending money unnecessarily on structures that soon must give place to better ones. Action should have been taken long ago and the city made respectable by the erection of properly constructed accessible stations.

---

THE extreme heat of the present summer that has pervaded the whole country has been trying in Buffalo for many days; but with an extreme temperature of 91° and rarely going above 90°, we have less to complain of than most of the large cities. Fortunately, no lives have been lost up to the present writing resultant from heat stroke. One of the commendable innovations introduced this season is hats for horses which have now come into common use for work teams. A wet sponge on the top of the head is a great relief to a work horse and undoubtedly has saved the lives of many useful animals. The Erie County Society for the Prevention of Cruelty to Animals and the veterinary surgeons agree in endorsing the utility of this valuable protection to animals. The hats are very inexpensive, costing 35 cents.

---

DR. DANIEL LEWIS, state commissioner of health, has secured a building in Albany for the establishment of a state antitoxin laboratory. Dr. H. D. Pease, of the Sheffield Scientific School, Yale University, has been appointed director of the new laboratory. The animal house, it is expected, will be provided with the most perfect hygienic conditions attainable for such purposes and will be supplied with about 15 horses for the manufacture of serum. Dr. Pease will be assisted in the work by five trained assistants. In discussing the subject recently, Dr. Lewis said:

The importance of this laboratory to the people of the state can hardly be overestimated. The commissioner of health proposes to supply state institutions with diphtheria and other antitoxins free of cost and also furnish these remedies to municipalities which are not already provided with a similar laboratory, for parents who are unable to buy them.

Antitetanus and antityphoid serums will also be manufactured in the laboratory. Health officers throughout the state can now secure from the state department examination of cultures from diphtheria patients, thus verifying a diagnosis as well as demonstrating when a diphtheria patient has so far recovered as to be allowed out of quarantine.

The sputum from cases of pulmonary tuberculosis is also examined by Dr. Blumer, director of the bureau of pathology and bacteriology of the department. These new departments, therefore, are intended to supply to all the health officers throughout the state the same facilities for investigation, diagnosis and treatment of infectious diseases as are now supplied by the city of New York.

---

The Ohio State Board of Medical Registration and Examination has obtained from the Supreme Court of Ohio an important decision which establishes its right to determine whether a medical college is in good standing. The board threw out the diplomas of the Hygea Medical College of Cincinnati, whereupon the college applied for a writ of mandamus compelling such recognition. The court sustained the board, the decision in effect being that a writ of mandamus will not issue, on the relation of a medical college, to compel the State Board of Medical Registration and Examination to recognise the college as a medical institution in good standing, nor to compel the board to issue certificates to practise medicine in that state, to holders of diplomas from such college. Decided March 26, 1901.

This decision will establish the Ohio board on a firm foundation, and the precedent thus created will strengthen the hands of boards in other states.

